# Pan-PCR Diagnostic Efficacy in Comparison with Traditional Methods in Patients with Septic Arthritis

**DOI:** 10.30699/ijp.2025.2058193.3456

**Published:** 2025-11-11

**Authors:** Erta Rajabi, Kousha Farhadi, Malihe Hasannezhad, Mahsa Azadbakhsh, Alireza Abdollahi, Seyed Hadi Kalantar, Sara Ghaderkhani

**Affiliations:** 1 School of Medicine, Tehran University of Medical Sciences, Tehran, Iran; 2 Department of Infectious Disease and Tropical Medicine, School of Medicine, Imam Khomeini Hospital Complex, Iranian Research Center for HIV/AIDS, Iranian Institute for Reduction of High-Risk Behaviors, Tehran University of Medical Sciences, Tehran, Iran; 3 Department of Pathology, School of Medicine, Imam Khomeini Hospital Complex, Tehran University of Medical Sciences, Tehran, Iran; 4 Department of Orthopedics, School of Medicine, Joint Reconstruction Research Center, Imam Khomeini Hospital Complex, Tehran University of Medical Sciences, Tehran, Iran

**Keywords:** Culture, Arthritis, Infectious, Polymerase Chain Reaction

## Abstract

**Background & Objective:**

Septic arthritis is an emergent condition caused by an infection of the joint synovial fluid. If left untreated, it can lead to irreversible damage to the affected joint. Our study focused on providing a concise profile of the Iranian population and the diagnostic roles of synovial pan-PCR and culture.

**Methods:**

In an observational study, we evaluated the characteristics of all patients diagnosed with septic arthritis and admitted to a teaching center hospital complex. We extracted and analyzed the study of population's demographics and laboratory values.

**Results:**

This study included 50 patients diagnosed with septic arthritis. 56% of our study population were male, and the mean age was 50.48. There were significant associations between synovial WBC counts and positive synovial culture results. Further comparison of the two diagnostic methods revealed higher pan-PCR accuracy than synovial culture.

**Conclusion:**

Pan-PCR may have higher diagnostic accuracy than synovial culture in hospitalized patients with septic arthritis.

## Introduction

Septic arthritis (SA), or the joint synovial fluid and surrounding tissue infection, is an infectious, orthopedic, and rheumatologic emergency requiring prompt attention and intervention (1-3). It most commonly affects prosthetic joints, but native joint SA, though rare, is possible (4,5), with an approximate incidence of 2–10 cases per 100,000 people annually (3). This incidence is expected to rise due to aging and increasing comorbidities (4). The absence of a proper basement membrane layer within the synovium makes it a vulnerable target for viral, bacterial, and fungal pathogens (5), whether through direct inoculation, hematogenous spread, postoperative contamination, or infected adjacent soft tissue (6,7). The most commonly reported affected joints within the literature are knees, hips, elbows, shoulders, and wrists (7,8). Although the nature of the microorganisms responsible for SA is highly host-dependent, the most common microorganisms include *Staphylococcus aureus* and *Streptococcus spp.* (9,10). Currently, diagnosis is determined by combining the physician's clinical decision, laboratory data, imaging, and a confirmed synovial fluid culture (11,12). Clinical features include erythema, swelling, and joint tenderness, as well as decreased range of motion in the affected joint, malaise, and fever (13). Synovial fluid culture PCR (polymerase chain reaction) sensitivity has been estimated to be about 70% (14,15). 

The field has been revolutionized by PCR-based techniques, which are now widely used in the diagnosis of a wide variety of infectious diseases, including SA, with pan-PCR considered a superior option compared to conventional PCR, due to its broader ability to detect diverse bacterial and fungal microorganisms, reduced primer bias, and improved detection of difficult-to-culture pathogens (15, 16). Despite the available data on the characteristics of those with septic arthritis in other countries, Iran needs comprehensive data on demographics, characteristics, and the most common pathogens responsible for this condition. Our study aimed to provide a concise view of patients with SA to evaluate the diagnostic accuracy of pan-PCR in comparison with synovial culture.

## Materials and Methods

In an observational study, we evaluated the characteristics of 50 patients diagnosed with septic arthritis who were admitted to a tertiary teaching hospital complex in Tehran, Iran.

### Study Design

Between 2023 and 2024, 50 patients diagnosed with SA admitted to a tertiary teaching center hospital complex were included in our study. The Hospital Information System (HIS) was used to retrieve demographic data, details about the affected joint, comorbidities, laboratory tests' results, synovial fluid culture, pan-PCR, and reported pathogens after selecting the target population. The inclusion criteria were all individuals with diagnosed SA who underwent diagnostic synovial fluid analysis for arthrocentesis. Patients with clear synovial fluids, normal viscosity, and a joint white blood cell (WBC) count lower than 20000 (polymorphonuclear or PMN dominancy) were excluded from this study. Concurrently, all patients underwent PCR and culture diagnostic methods for direct comparison of their diagnostic performance.

### Ethical Considerations

This study was approved by the Research Ethics Committee, and patient confidentiality was maintained through coding and followed all relevant guidelines and regulations in accordance with the Declaration of Helsinki and the ethical standards of the responsible committee on human experimentation (Ethics code: IR.TUMS.IKHC.REC.1400.164).

### Sample Size Calculation

A standard formula for estimating proportions was used to determine the sample size: 

$n=\frac{z^{2}\times \overset{\text{\textasciicircum{}}}{p}\left(1-\overset{\text{\textasciicircum{}}}{p}\right)}{\varepsilon^{2}}$

*where Z* = 1.96 for 95% confidence, *p* = 0.5 as a conservative estimate of the proportion, and *d* = 0.14 representing the margin of error (14%), resulting in the calculated required sample size of 49. The relatively large margin of error was chosen due to practical constraints in recruiting individuals diagnosed with SA. 

### Statistical Analysis

Descriptive data were reported as frequency and percentage, while scale variables were reported as mean and standard deviation (SD). To evaluate the differences between different groups, the chi-square test for nominal variables and the Mann-Whitney U test for scale variables were utilized. Moreover, to accurately compare the two diagnostic methods, McNemar's chi-squared test was performed. Sensitivities for each test were calculated as the proportion of true positive cases identified by the method divided by the total number of confirmed cases.

**Table 1:** Demographics of the study population and results of chi-square analysis

P value	Positive culture, n (%)	P value	Positive Pan-PCR, n (%)	Percentage	Total		
<0.001	18 (64.3)	0.004	19 (67.9)	56	28	Male	
1	1 (33.3)	1	1 (33.3)	6	3	Previous joint disease	
0.190	9 (47.4)	1	10 (52.6)	38	19	Diabetes mellitus	
0.283	0 (0)	0.609	1 (25.0)	8	4	Malignancy	
1	1 (33.3)	1	1 (33.3)	6	3	Recent antibiotic use	
0.494	5 (45.5)	1	6 (54.5)	22	11	Previous trauma	
1	1 (50.0)	1	1 (50.0)	4	2	Previous joint injection	
0.239	7 (26.9)	0.258	11 (42.3)	52	26	Swelling	
1	1 (25.0)	0.609	3 (75.0)	8	4	Right hip	
0.050	4 (80.0)	0.050	5 (100.0)	10	5	Left hip	
0.610	6 (31.6)	0.382	8 (42.1)	38	19	Right knee	
0.366	5 (26.3)	0.145	7 (36.8)	38	19	Left knee	
0.336	3 (60.0)	0.349	4 (80.0)	10	5	Left shoulder	
1	1 (50.0)	0.490	2 (100.0)	96	48	Monoarticular	
				50	25	Positive synovial PCR	
				36	18	Positive synovial culture	
				6	3	MRSA	Isolated microorganism
				8	4	MSSA	
				14	7	*E. coli*	

## Results

Within our study population, the most reported mean age (±SD) was 50.48 (±16.30) years. A male dominance trend was observed among our study population. (n=28, 56%) Diabetes mellitus (n = 19, 38%) and previous trauma to the affected joint (n = 11, 22%) were the two most commonly reported predisposing factors, and the majority were diagnosed with monoarticular SA. (n = 48, 96%). Four (4%) patients reported a positive history of intra-articular injections. The knee was the most commonly affected joint, with equal distributions between the right and left knees (n = 19, 38%), followed by five cases of left hip and left shoulder involvement. All patients reported tenderness, erythema, and decreased range of motion. 

Thirty-six percent (n = 18) of PCR and synovial fluid cultures were positive concurrently, and synovial fluid PCR and culture were positive in 25 and 18 cases, respectively, yielding sensitivities of 50% for PCR and 36% for culture. The most commonly reported microorganism was *E. coli *(*Escherichia coli*) (14%), followed by *MSSA* (*methicillin-sensitive Staphylococcus aureus*) (8%) and *MRSA* (*methicillin-resistant Staphylococcus aureus*) (6%). The mean (SD) synovial fluid WBC counts were 29869. 40±16987.84, and the mean (SD) blood WBC cells/mm^3^ were 10260±3608.32. Synovial WBC counts were significantly higher among those with positive PCR and culture results. (P values = 0.029 and <0.001, respectively.) The mean (SD) CRP and ESR counts were 94.52±47.96 and 80.28±31.06, respectively.

Table 3 illustrates the McNemar's test, performed to compare the accuracy of the two employed diagnostic tests. As shown, pan-PCR revealed higher efficacy compared to the synovial culture test in the same population. There was a point probability of 0.008, and a significant difference between the two diagnostic results was found (P value: 0.016).

**Table 2:** Mean, SD, and independent sample T test's results of continuous variables

P value	Positive culture		P value	Positive Pan-PCR		SD	Mean	
	SD	Mean		SD	Mean			
0.426	13.96	48.00	0.242	14.74	47.76	16.305	50.48	Age
<0.001	13297.587	40236.11	0.029	16271.94	35068.40	16987.84	29869.40	Synovial WBC*
0.534	27.04	85.00	0.529	62.27	95.29	48.71	90.76	Synovial glucose
0.425	32.33	123.89	0.469	34.07	122.28	33.14	118.84	FBS*
0.205	4470.83	11238.89	0.665	4054.18	10484.00	3608.32	10260.00	Blood WBC
0.571	44.36	99.72	0.418	48.58	88.96	47.96	94.52	CRP*
0.941	30.45	80.72	0.740	28.49	78.80	31.06	80.28	ESR*

^*^^WBC: white blood cell, FBS: fasting blood sugar, CRP: C-reactive protein, ESR: erythrocyte sedimentation rate ^

**Table 3:** Comparison between synovial pan-PCR and culture using McNemar chi-squared test

		Synovial PCR		McNemar Test	
		Negative results N (%)	Positive results N (%)	Point probability	P value
Synovial culture	**Negative results**	25 (100)	7 (28.0)	0.008	**0.016**
	**Positive results**	0 (0)	18 (72.0)		

## Discussion

*Septic arthritis *is an emergent diagnosis that affects one or many joints. In our study, we demonstrated the characteristics of patients diagnosed with septic arthritis admitted to a teaching hospital center in Iran. Our study population consisted mainly of males with a mean age of 50.48 who predominantly presented with erythema, decreased range of motion, and tenderness in the affected joint. Additionally, a higher diagnostic accuracy of synovial pan-PCR when compared to the synovial culture results of the same population was observed*.*

Similar to the literature, the most commonly affected joints within our study were the right and left knees (17). Ernst et al. conducted a study to evaluate the diagnostic efficacy of ESR and CRP in septic arthritis. In their study, the knee and male populations were predominant, with a lower mean age than that reported in ours. The mean WBC, CRP, and ESR counts were lower than those reported in our study. Additionally, they identified CPR, in contrast with ESR, as a favorable diagnostic laboratory test in patients with SA (18). Fottner et al. reported results similar to those of Ernst et al. Mean (±SD) CRP and WBC levels within their study population were reported as 15.03±5.73 and 11.31±3.34, respectively (19). The study population differences and the stage of septic arthritis disease at the time of diagnosis could explain this difference in CRP and WBC levels. 

Li et al. reported 72 patients with septic arthritis and evaluated the sensitivity of blood WBC, ESR, and joint WBC. The majority of their study population consisted of men with a mean age of 52, and their most common comorbidity was diabetes mellitus. The most commonly affected joints were the knees and shoulders, and the most commonly reported microorganism was Staphylococcus aureus. Within their study population, the mean WBC, ESR, and joint WBC were 12700 cells/mm^3^, 103 mm/hr, and 114000 cells/mm^3^. Their study population was similar to ours when comparing demographics, and both blood and joint WBC reported in their study were higher than ours. We also reported significant differences in the amount of joint WBC based on synovial culture results (20). In a separate study on synovial fluid leukocyte counts in patients with total knee arthroplasty failure, 72 patients with prosthetic joint infection were included. Synovial WBC counts, PMN counts, and positive synovial culture results were significantly higher in their study (21).

Regarding the diagnostic accuracy of PCR compared to synovial culture, one study reported on the superiority of synovial PCR test results for detection of low-virulent bacteria (22). This finding is in accordance with ours, where pan-PCR showed higher diagnostic accuracy.

We would like to acknowledge the limitations in our study. Firstly, our hospital admitted a limited number of patients diagnosed with SA, and a study with a larger population is warranted to provide a concise view of the Iranian population diagnosed with SA. Second, because this study is retrospective, there may be some bias in terms of memory and data collection, which could affect the results. Thirdly, the absence of a control group limited this study’s ability to assess false-positive rates of the diagnostic methods and specificities.

## Conclusion

In this study we reported the superiority of synovial pan-PCR compared to synovial culture, with sensitivities of 50% and 36%, respectively. The results of this study could be used to improve the timely diagnosis of those affected by septic arthritis.

## Data Availability

The data supporting the results of this study are available upon request from the corresponding author.
